# Botulinum Toxin for Essential Tremor and Hands Tremor in the Neurological Diseases: A Meta-Analysis of Randomized Controlled Trials

**DOI:** 10.3390/toxins14030203

**Published:** 2022-03-10

**Authors:** Yen-Hui Liao, Chien-Tai Hong, Tsai-Wei Huang

**Affiliations:** 1 Department of Neurology, Shuang-Ho Hospital, Taipei Medical University, New Taipei City 235, Taiwan; greydove777@gmail.com (Y.-H.L.); chientaihong@gmail.com (C.-T.H.); 2 Department of Neurology, School of Medicine, College of Medicine, Taipei Medical University, Taipei 110, Taiwan; 3 School of Nursing, College of Nursing, Taipei Medical University, Taipei 110, Taiwan; 4 Cochrane Taiwan, Taipei Medical University, Taipei 110, Taiwan; 5 Center for Nursing and Healthcare Research in Clinical Practice Application, Wan Fang Hospital, Taipei Medical University, Taipei 110, Taiwan; 6 Department of Nursing, Wan Fang Hospital, Taipei Medical University, Taipei 110, Taiwan

**Keywords:** botulinum toxin, tremor, randomized controlled trial, meta-analysis

## Abstract

Tremor is a common movement disorder. Essential tremor (ET) is the most common etiology of tremor, while hands tremor is the most disabling type of tremor. This study aimed to explore the effects of Botulinum toxin (BoNT) on tremor within 6 weeks of treatment, and the muscular weakness adverse effect within 6 weeks specifically in randomized controlled trials. PubMed, Embase, and Cochrane Library databases were searched. Tremor severity and grip strength after BoNT treatment were investigated. BoNT significantly attenuated hand tremor severity in patients with either essential tremor (ET), Parkinson’s disease or multiple sclerosis (Standardized mean difference [SMD] = −0.59, 95% confidence interval [CI], −0.95 to −0.24, *p* = 0.001, I^2^ = 46%). Regarding people with ET, BoNT significantly reduced their tremor severity, including hands tremor and head tremor within 6 weeks of treatment (SMD = −0.58, 95% CI, −0.28 to −0.88, *p* = 0.002, I^2^ = 0%). Electromyography (EMG) but not anatomical guidance BoNT injection provided significant benefit on the relief of tremor in both conditions. The principal adverse event was weakness, but it did not worse within 6 weeks of BoNT treatment (SMD = −0.35, 95% CI, −0.83 to 0.12, *p* = 0.07, I^2^ = 57%), as assessed by the subjective grip strength. In conclusion, BoNT was an effective treatment for the hand tremor and ET, and EMG guidance injection was preferred. In addition, the muscular weakness adverse effect was not significant.

## 1. Introduction

Tremor is defined as an involuntary, rhythmic, and oscillatory movement of a body part [1]. Although neurological diseases such as stroke, Parkinson’s disease (PD), and multiple sclerosis (MS) also result in tremor, essential tremor (ET) is the most common among tremor and movement disorders [2]. Tremor has numerous clinical manifestations, depending on the frequency, region of the body affected (e.g., arm, voice, head), association with body movement (e.g., resting or action), and anatomical location of brain lesion (e.g., basal ganglia, cerebellum) [3]. Tremor, especially hand tremor, interferes with the activities of daily life and may lead to social awkwardness or impaired quality of life [4].

The prevailing treatment strategy for tremors involves medication and surgical intervention. Medications such as propranolol, antiseizure medications, antidepressants, and benzodiazepines offer partial benefits but also some adverse effects [5,6]. For instance, although the treatment response to propranolol is remarkable in some patients [7], its adverse effects, including hypotension, bradycardia, depression, and fatigue, may be intolerable, especially for older patients; severe bradycardia with syncope has even been reported [8]. Some antiseizure medications and benzodiazepines are effective for tremor treatment, but the main side effect, sedation, usually results in the discontinuation of medications [8]. Surgical intervention may include deep brain stimulation (DBS) or magnetic resonance-guided, focused ultrasound (MRgFUS) thalamotomy. DBS has demonstrated robust therapeutic effects [9,10,11,12]; however, possible DBS-related complications, including intracranial and intracerebral hemorrhage, infection, and improper DBS location, have been noted [13]. DBS in the ventral intermediate nucleus of the thalamus may be associated with stimulation-induced paraesthesia, dysarthria, and ataxia [14]. Other minor side effects such as paresthesia, headache, dysarthria, and dyskinesia have also been reported [10,13,15,16]. Moreover, the invasive nature of DBS affects patients’ willingness to undergo treatment. The MRgFUS thalamotomy is a novel, noninvasive alternative [17]. However, the cost and potential disequilibrium side effects sensation limit this treatment’s utility [18].

Botulinum toxin (BoNT) is a potent biological toxin produced by Clostridium botulinum and related species. BoNT prevents the release of the neurotransmitter acetylcholine from axon endings at the neuromuscular junction, which may cause lethal paralysis. The effectiveness of BoNT attracts intense interest in clinical and scientific research, and the toxin has emerged as one of the most versatile therapeutic agents in modern medicine [19]. The therapeutic application of BoNT has expanded rapidly since December 1989, when it was first approved by the US Food and Drug Administration (FDA) for the treatment of strabismus, blepharospasm, and other facial nerve disorders, including hemifacial spasm. The long-term efficacy and safety of BoNT in the treatment of dystonia and other movement and neurologic disorders have since been demonstrated. Despite the well-known adverse effect of reversible weakness, BoNT overdose-related lethal respiratory paralysis was extremely rare [20,21].

BoNT is an alternative in the treatment of refractory ET cases [22]. However, evidence supporting such a strategy is limited, and the results of randomized controlled trials (RCTs) regarding the therapeutic effects of BoNT in the treatment of tremors have been inconsistent [23,24,25,26,27,28,29,30]. By contrast, the side effects of BoNT, in particular, muscle weakness, are not well understood, limiting its feasibility for treating tremors. This study investigated both the beneficial and adverse effects of BoNT in the treatment of tremors through a meta-analysis of published RCTs.

## 2. Results

**Figure 1:**
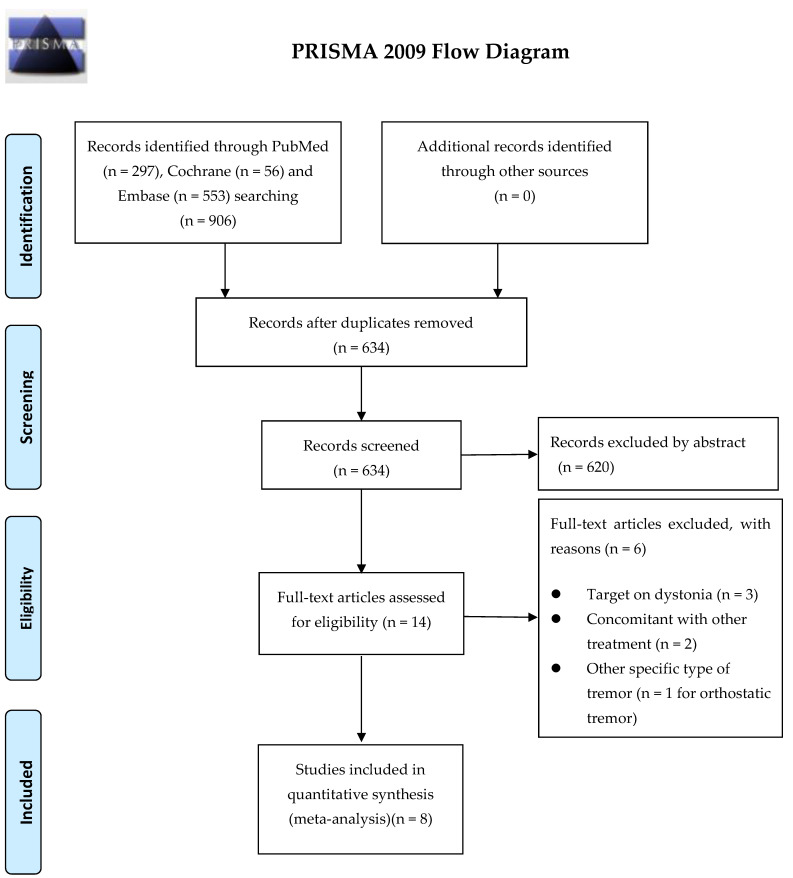
Flowchart of the literature search.

Figure 1 presents a flowchart of study screening and selection. The search returned studies published before December 2021. Among 906 publications retrieved in the initial search, 272 were excluded because they were duplicates, and 620 were excluded because they were not RCTs. The full texts of the remaining 14 studies, all of which were RCTs, were accessed. Six of those were excluded—three were focused on dystonia, two involved another concomitant treatment, and one targeted orthostatic tremor. The remaining eight studies all investigated the effects of BoNT therapy in patients with tremors, specifically ET or hand tremors resulting from various neurological diseases, and were included in the quantitative analysis [23,24,25,26,27,28,29,30]. The sample sizes of these studies, which had a slight male predominance, ranged from 25 to 87 participants. The interventions were performed through intramuscular injection of BoNT according to the location of the tremor. The total BoNT administered ranged from 35 to 120 units. Table 1 lists the characteristics of the included studies. Five studies focused on ET patients and assessed the outcome (hand or head tremor) within 6 weeks. Three of these five ET studies also assessed the outcome between 7 and 12 weeks after the treatment. Regarding hand tremors, all eight studies investigated the treatment response within 6 weeks, and six of these studies also presented the treatment outcome between 7 and 12 weeks. The various outcome assessments used were the Bain composite tremor score, Section 16 (tremor subitem) of the Unified Parkinson Disease Rating Scale (UPDRS), subjective assessment by an investigator, the Fahn–Tolosa–Marin Part B motor performance score, and the Patient Global Impression of Change score. Risk-of-bias assessment suggested that all eight studies were low risk or had some bias concerns (Supplementary Table S1).

### 2.1. Effects in Essential Tremor

**Figure 2:**
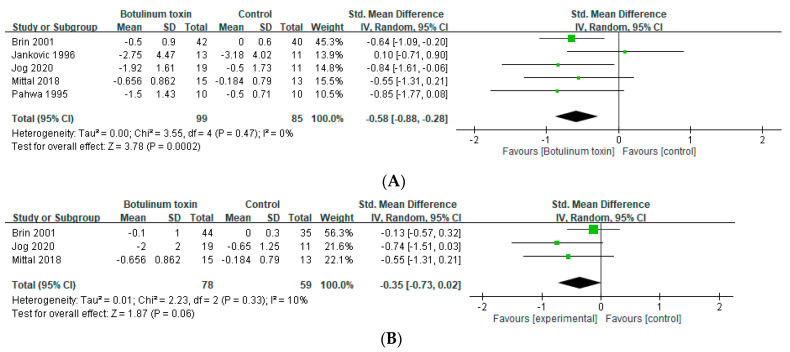
Effects of botulinum toxin on tremor in patients with essential tremor (**A**) within 6 weeks and (**B**) with electromyography guidance.

Five trials assessed the short-term effects in the severity in the patients with ET within 6 weeks [24,25,26,28,29]. BoNT treatment significantly attenuated the severity of tremor in patients with ET in the short term (SMD = −0.58, 95% CI, −0.28 to −0.88, *p* = 0.002, I^2^ = 0%: Figure 2A). Three of five trials assessed the effects between 7 and 12 weeks [24,26,28]; however, that significant benefit was not maintained in the long term (SMD = −0.35, 95% CI, −0.73 to 0.02, *p* = 0.33, I^2^ = 10%; Supplementary Figure S1). BoNT treatment with electromyography (EMG) guidance, three of the five studies [24,28,29] demonstrated significant improvement of the tremor severity within 6 weeks. (SMD = −0.65, 95% CI, −1.01 to −0.30, *p* = 0.0003, and I^2^ = 0%; Figure 2B). However, there was no significant improvement reported in the two studies [25,26] regarding anatomical guidance within 6 weeks (SMD = −0.38, 95% CI, −1.29 to 0.54, *p* = 0.10, and I^2^ = 63%; Supplementary Figure S2).

### 2.2. Effects in Hands Tremor

**Figure 3:**
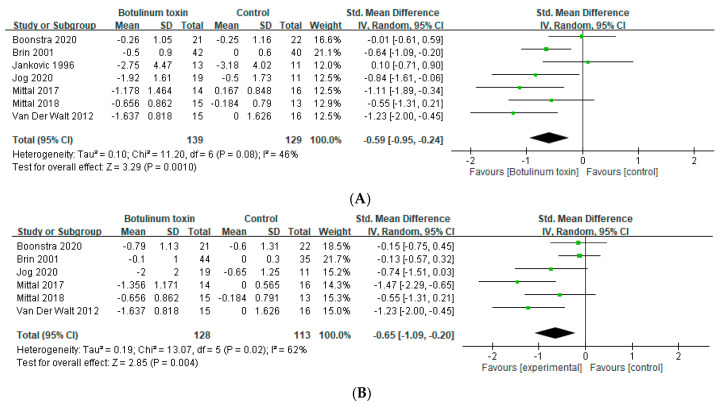
Effects of botulinum toxin on hand tremor (**A**) within 6 weeks and (**B**) with electromyography guidance.

Seven trials [23,24,25,26,27,28,30] assessed the effects of BoNT on the severity of hand tremor within 6 weeks. BoNT treatment was significantly effective in relieving the severity of hand tremor in the short term (SMD = −0.59, 95% CI, −0.95 to −0.24, *p* = 0.001, I^2^ = 46%; Figure 3A). Six of them also assessed the effect between 7 and 12 weeks, which also demonstrated a significant benefit (SMD = −0.65, 95% CI, −1.09 to −0.20, *p* = 0.004, I^2^ = 62%; Supplementary Figure S3) [23,24,25,26,27,30]. Five trials [23,24,27,28,29,30] used the EMG guidance, and significant reliving of hands tremor was noted in the group with EMG guidance (SMD = −0.66, 95% CI, −1.07 to −0.25, *p* = 0.002, and I^2^ = 50%; Figure 3B). The result of anatomical guidance treatment was identical with the aforementioned due to the same origin of data (both studies evaluated hands tremor in ET patients) (Supplementary Figure S2).

### 2.3. Muscle Weakness

**Figure 4:**
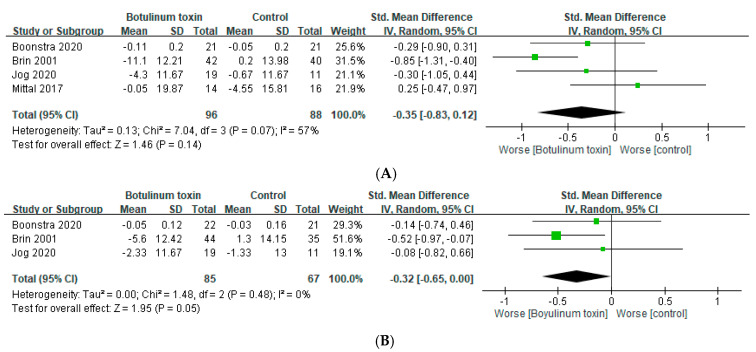
Adverse effects of botulinum toxin on grip strength (**A**) within 6 weeks and (**B**) between 7 and 12 weeks.

Muscle weakness is the most common and concerning adverse effect of BoNT. Some of the included RCTs assessed handgrip strength subjectively as a parameter of weakness. In four trials, it was assessed within 6 weeks [23,24,26,27], and in three of the four trials, it was also evaluated between 7 and 12 weeks [23,24,26]. BoNT treatment did not significantly weaken handgrip in either the short term (SMD = −0.35, 95% CI, −0.83 to 0.12, *p* = 0.07, and I^2^ = 57%; Figure 4A) or long term (SMD = −0.32, 95% Cl, −0.65 to 0.00, *p* = 0.48, and I^2^ = 0%; Figure 4B). However, weakness at the injection site during administration was reported. Mild hand weakness was reported in some cases in nearly all of the included RCTs. Some patients even discontinued BoNT treatment because of muscular weakness, but most of the patients tolerated mild weakness. Swallowing difficulties were reported in head tremor trials. In addition to weakness, pain at the injection site was observed. Other adverse effects, including hematoma, paresthesia, stiffness, and cramping, were reported.

## 3. Discussion

The present study revealed that BoNT treatment significantly attenuated the severity of ET and hands tremor within 6 weeks after treatment, especially with the assistance of EMG guidance. In general, the primary adverse effect, hand muscle weakness, was tolerable, and BoNT did not result in significant weakness, as assessed by objective measurement. The present meta-analysis provides strong evidence for the management of tremors with BoNT and eases concerns regarding the adverse effects, specifically weakness. 

BoNT A and B successfully treat a variety of involuntary movement disorders. At present, four BoNTs are widely available: three type A toxins (onabotulinum, incobotulinum, and abobotulinum toxin A) and one type B toxin (rimabotulinum toxin B) [31]. Since its initial approval in 1989 by the US FDA for the treatment of blepharospasm and other facial spasms, BoNT has evolved into a therapeutic modality for a variety of neurological and nonneurological disorders [32], and its use in tremor treatment can be traced back as far as 1981 [33]. Since that time, several studies have reported divergent results regarding the response of tremor to BoNT. Recently, a systematic review of BoNT treatment of tremors determined that most previous studies were open-label and that well-designed RCTs assessing the utility of BoNT in the treatment of ET and Parkinson’s disease (PD) tremor are necessary [34]. Due to the ineffectiveness of other medical treatments in some patients and the risks involved with surgical intervention, BoNT is potentially appropriate for managing medically refractory and surgery-contraindicated tremors. This meta-analysis supports claims of BoNT treatment’s significant therapeutic effect of alleviating tremor severity; therefore, BoNT can provide an alternative treatment option for patients with severe and debilitating tremor disorders. 

Due to the biochemical nature of BoNT, the therapeutic effects of BoNT persist for approximately up to 12 weeks [35]. Different types of BoNT also have various effect durations. In the present study, the type of BoNT administered varied among the included RCTs. Two of the RCTs used onabotulinum, and another two used incobotulinum, both of which are type A BoNTs. One RCT did not mention the specific type of BoNT employed. The activity of BoNT A and B lasted for 6–8 weeks, with the maximal benefit occurring 4–6 weeks after injection [31]. In this study, a cutoff point of 6 weeks was used to distinguish the short and long terms. The short-term effects directly resulted from the neuromuscular junction blockade of BoNT, whereas the long-term effects may be caused by reciprocal changes at the upstream level, including the central nervous system, a principle that was demonstrated in the treatment of pain [36].

Weakness is one adverse effect of BoNT and may result in the discontinuation of BoNT treatment. Although this side effect is rare, it caused several study participants to discontinue treatment [37]. Mittal et al. reported that mild weakness may persist for 4 weeks. A customized, flexible injection was suggested to reduce the risk of weakness, compared with a fixed injection [28]. In the present meta-analysis, the assessment of handgrip strength as a subjective measurement of muscle power revealed no significant weakness effect resulting from BoNT treatment. However, a considerable weakness effect has been reported in studies administering BoNT treatment for voice tremor [37] or primary orthostatic tremor [38]. The side effect of swallowing difficulty was noted in one included study [29]. Thus, the dose and safety of BoNT injections should be monitored carefully, especially in the treatment of voice or neck tremors. Another finding from the included studies of the present meta-analysis is that only Brin et al. had a fixed protocol for BoNT injection, while the other four studies used a customized, flexible injection [23,24,26,27], which correspond with the content in a previous article [28]. Further studies are required to define the safe dosage range for individual muscles, especially for neck muscles, and thereby reduce the risk of subsequent weakness. The suggestion of dosage in different parts with tremors was mentioned in Kamel JT et al. [39], which may be used as a protocol in the future for further RCTs.

Several factors contributed to the heterogeneity in the present meta-analysis. The present study included RCTs covering the treatment of tremors from different neurological diseases, such as MS, PD, and ET. Male participants predominated all included RCTs, except one study that focused on the treatment of MS-induced tremor. Moreover, the BoNT dosage and treatment protocol in each RCT varied, and a second dose of BoNT was issued in some of the RCTs. Due to these factors, the heterogeneity was remarkable for the analysis of BoNT treatment effects on hand tremors. Regarding ET, however, the heterogeneity was low, and the treatment response was consistent in the short term.

The present study has some limitations. First, several included studies had small sample sizes, which might represent only a specific group of patients. This study mitigated this shortcoming by employing a meta-analysis design. Second, the present study only focused on one type of tremor (hand tremor) and one tremor disorder (ET), and this focus may limit the application of the results to other types of tremor disorder. Finally, the assessment methods were diverse, including the UPDRS (tremor subitem), Bain composite tremor score, and Patient Global Impression of Change scale, which all assess tremor with different criteria. The evaluation results also varied on the basis of the investigators’ observations, primarily because of the lack of a unified model for subjective tremor assessment. In future studies, surface electromyography is a possible means of subjectively assessing tremors [23]. Moreover, the fragmented information nature of meta-analysis is also an inevitable limitation of the present study [40]. Lastly, the effect and adverse effects of BoNT treatment are highly individualized, and in real-world experience, it usually takes 2–3 treatments for optimization. These tests are not allowed in the RCTs, which may underestimate the benefit of BoNT.

## 4. Conclusions

The present meta-analysis demonstrates that BoNT relieves tremor severity in patients with ET in the short term and in patients with hand tremor (resulting from different neurological diseases), in both the short and long term. The critical adverse effect of muscle weakness was not significantly associated with BoNT treatment in the included studies, and recorded cases were usually tolerable. Due to the low risk of systemic adverse effects and their reversible nature, BoNT is an ideal treatment option for medically refractory and debilitating tremors.

## 5. Materials and Methods

### 5.1. Inclusion Criteria

RCTs comparing the effects of various BoNT treatments on patients with tremors were screened. RCTs that clearly reported the inclusion and exclusion criteria for patients with tremors, protocols of BoNT therapy, and methods through which tremor severity was evaluated before and after the intervention were included. This study was registered on PROSPERO, a prospective international register of meta-analyses, under the file number CRD42021234537 (approval date 3 March 2021).

### 5.2. Search Strategy and Study Selection

Relevant RCTs published before December 2021 were identified from the PubMed, Embase, and Cochrane Library databases by using the following keywords and search strategy: (“tremor” (Title/Abstract) OR “tremors” (Title/Abstract)) AND (“botulinum A” (Title/Abstract) OR “botulinum toxin” (Title/Abstract) OR “botulinum neurotoxin B” (Title/Abstract) OR “botulinum toxin type A” (Title/Abstract) OR “botulinum toxin type B” (Title/Abstract) OR “abobotulinumtoxinA” (Title/Abstract) OR “onabotulinumtoxinA” (Title/Abstract) OR “incobotulinumtoxinA” (Title/Abstract) OR “rimabotulinumtoxinB” (Title/Abstract)). Only English publications were considered. 

### 5.3. Data Extraction

The baseline and outcome data were independently retrieved by two reviewers (Chien-Tai Hong and Yen-Hui Liao). Data on study designs, participant characteristics, eligibility criteria, and interventions were also extracted. The data were recorded individually by the reviewers and compared, and any disagreements were resolved by a third reviewer (Tsai-Wei Huang).

### 5.4. Appraisal of Methodological Quality

Two reviewers (Chien-Tai Hong and Yen-Hui Liao) independently assessed the methodological quality of each study by using the revised Cochrane risk-of-bias tool version 2.0 (Cochrane, Oxford, UK). Specifically, eight RCTs were assessed with the tool, which classifies a study’s risk of bias as high risk, low risk, or “some concerns” on the basis of the potential for biases involving the randomization process, deviations from intended interventions, missing outcome data, outcome measurement, and the selection of reported results.

### 5.5. Outcomes

Since the effect of BoNT maximizes two weeks after the treatment and gradually wanes, most studies evaluated the effect at multiple time points, first between 4 and 6 weeks and then between 8 and 12 weeks. The primary outcome timing of the present meta-analysis was the effect within 6 weeks, while the secondary outcome was the effect during 7–12 weeks. Regarding the effect of BoNT on tremors, the main outcome was tremor scores. If a study employed more than one scale for investigating tremors, the more commonly used scale was selected for the outcome analysis. The adverse effect of grip strength loss, as assessed through subjective measurement, was also extracted for meta-analysis.

### 5.6. Statistical Analysis

Data were entered into Review Manager 5.4 software (Cochrane, Oxford, UK) and analyzed. The meta-analysis was performed in accordance with the Preferred Reporting Items for Systematic Reviews and Meta-Analyses statement. Standard deviations (SDs) were calculated from the provided confidence intervals (CIs), standard errors, and interquartile ranges. The effect sizes of the continuous outcomes are reported as standardized mean differences (SMDs) and 95% CIs. Pooled weighted mean differences (WMDs) were estimated using the DerSimonian and Laird random-effects model. Statistical significance was indicated by a *p* value of <0.05 or a 95% CI that did not include 1 for the relative risk or 0 for the WMD.

Statistical heterogeneity and inconsistency in treatment effects were evaluated using the Cochrane Q test (for which significance was set at *p* < 0.10) and the I^2^ statistic, respectively. Specifically, the I^2^ test was used to determine the proportion of total outcome variability attributable to heterogeneity.

## Figures and Tables

**Table 1:** Characteristics of included studies.

Author (Year)	Inclusion Criteria	No. of Patients (Male, %)	Age, Mean ± SD (y)	Treatment Target	Intervention	Outcome Measurement	Side Effect
Boonstra et al. (2020) [23]	RRMS, SPMS, or PPMS with unilateral hand tremor	43 (11, 25.6%)	E 45.9 ± 13.5 C 47.0 ± 7.55	Upper limbs	BT 100 U	Bain composite tremor score (0–10) at 6 and 12 weeks	Significant decrease in muscle strength
Brin et al. (2001) [24]	ET with bilateral postural hand tremor	133 (90, 68%)	68.5 ± 11.2	Upper limbs	Low dose: BT 50 U; high dose: BT 100 U	Subjective assessment by investigator compared with baseline (−4 to 4) at 6 and 12 weeks	Weakness, pain in injection site, stiffness, cramping, hematoma, and paresthesia
Jankovic et al. (1996) [25]	ET with moderate to severe tremor	25 (16, 64%)	E 56.2 ± 13.0 C 67.4 ± 12.4	Upper limbs	BT 50 U	Functional severity of tremor (0–4)	Mild and moderate finger weakness
Jog et al. (2020) [26]	ET with moderate to severe tremor	30 (15, 50.0%)	E 68.1 ± 10.6 C 68.2 ± 10.2	Upper limbs	Mean dose: BT 116.3 U	Fahn–Tolosa–Marin Part B motor performance score	Localized muscular weakness, dry mouth, and dysphonia
Mittal et al. (2017) [27]	PD with moderate to severe tremor	30 (23, 77%)	E 68.50 (range: 57–87) C 62 (range: 51–81)	Upper limbs	BT 85–110 U with follow-ups at 4 and 8 weeks	UPDRS Section 16 at 4 and 8 weeks	Weakness in some patients
Mittal et al. (2018) [28]	ET with moderate to severe tremor	28 (13, 46.2%)	E 70 (range: 43–81) C 62.5 (range: 25–82)	Upper limbs	BT 80–120 U	NIHCGC (0–4) at 4 and 8 weeks	Mild hand weakness (from a few days to 4 weeks)
Pahwa et al. (1996) [29]	ET with horizontal head tremor	10 (1, 10%)	65.9 (range: 50–82)	Head	BT 100 U	Subjective assessment involving baseline comparison (−3 to 3) at 4 and 8 weeks	Neck weakness, swallowing difficulty, headache, and dizziness
Van Der Walt et al. (2012) [30]	RRMS or SPMS with disabling hand tremor	23 (6, 26%)	49.6 ± 11.0	Upper limbs	BT 35–100 U	Bain composite tremor score (0–10) at 6 and 12 weeks	Weakness (resolved in 2 weeks)

RRMS: relapsing–remitting multiple sclerosis; SPMS: secondary progressive multiple sclerosis; PPMS: primary progressive multiple sclerosis; PD: Parkinson’s disease; ET: essential tremor; SD: standard deviation; E: experimental group; C: control group; BT: botulinum toxin; UPDRS: Unified Parkinson Disease Rating Scale; NIHCGC: National Institutes of Health Collaborative Genetic Criteria tremor severity score.

## Data Availability

The datasets used and/or analyzed during the current study are available from the corresponding author on reasonable request.

## Supplementary Materials

The following supporting information can be downloaded at: https://www.mdpi.com/article/10.3390/toxins14030203/s1, Figure S1: Effect of Botulinum toxin on tremor between 7–12 weeks after treatment in patients with essential tremor; Figure S2: Effect of Botulinum toxin on tremor in patients with essential tremor anatomical guidance; Figure S3: Effect of Botulinum toxin on hands tremor between 7–12 weeks; Table S1: Risk-of-bias assessment results.
