# Supplementary material for: Botulinum Toxin for Essential Tremor and Hands Tremor in the Neurological Diseases: A Meta-Analysis of Randomized Controlled Trials
Source: Toxins (Basel). 2022 Mar 10;14(3):203. doi: 10.3390/toxins14030203 (PMC8955110; doi:10.3390/toxins14030203)
Supplement: Supplementary file 1 [file toxins-14-00203-s001.zip › toxins-1617292 sp.pdf]

Supplementary Materials: Botulinum Toxin for Essential Tremor and Hands Tremor in the Neurological Diseases: A Meta-Analysis of Randomized Controlled Trials

Yen-Hui Liao, Chien-Tai Hong, and Tsai-Wei Huang

Table S1. Risk-of-bias assessment results.

Author, (Year)	Adequacy of randomization	Deviation from intended intervention	Missing outcome data	Measurement of outcomes	Selection of reported results	Overall risk of bias
Boonstra, et al., 2020 [23]	Low risk	Low risk	Low risk	Low risk	Low risk	Low risk
Brin, et al., 2001 [24]	Low risk	Low risk	Low risk	Low risk	Low risk	Low risk
Jankovic, et al., 1996 [25]	Low risk	Low risk	Some concerns	Low risk	Low risk	Some concerns
Jog, et al., 2020 [26]	Low risk	Low risk	Low risk	Low risk	Low risk	Low risk
Mittal, et al., 2017 [27]	Low risk	Low risk	Some concerns	Low risk	Low risk	Some concerns
Mittal, et al., 2018 [28]	Low risk	Low risk	Low risk	Low risk	Low risk	Low risk
Pahwa, et al., 1996 [29]	Some concerns	Low risk	Low risk	Low risk	Low risk	Some concerns
Van Der Walt, et al., 2012 [30]	Low risk	Low risk	Low risk	Low risk	Low risk	Low risk

a) It is likely that missingness depended on true value b) It is likely that missingness depended on true value
c) Not menetion allocation sequence concealed but not suggest a problem while baseline imbalances.

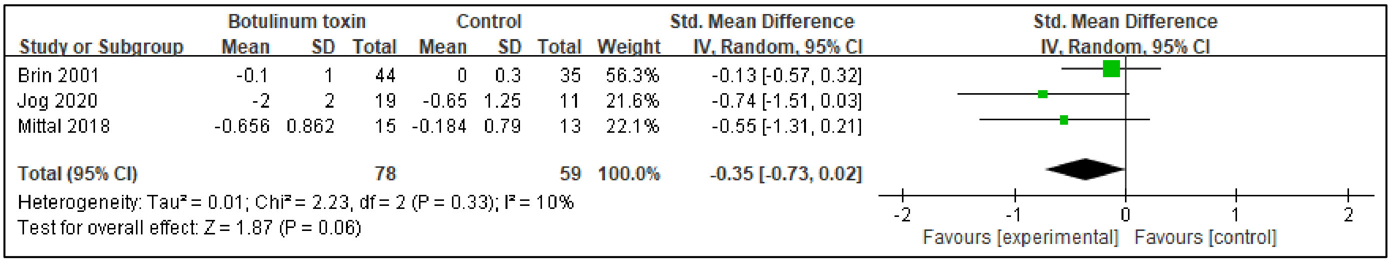

Figure S1. Effect of Botulinum toxin on tremor between 7–12 weeks after treatment in patients with essential tremor.

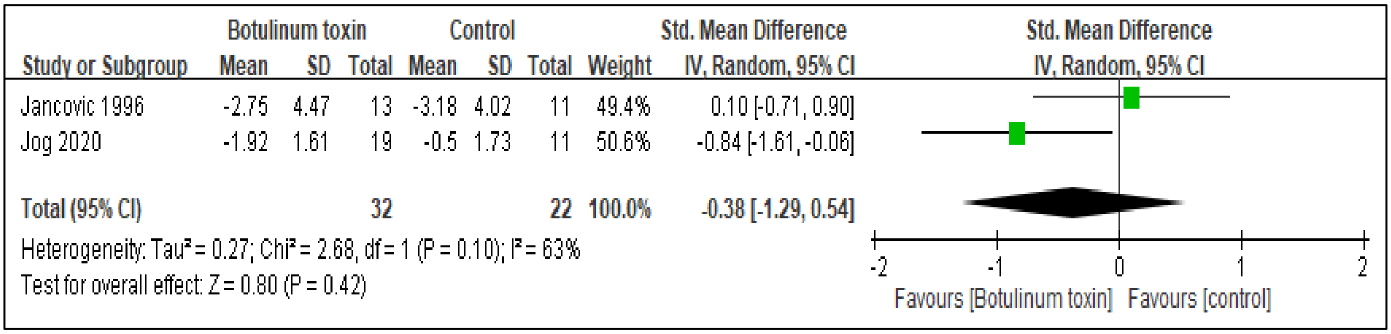

Study or Subgroup	Botulinum toxin Mean	SD	Total	Control Mean	SD	Total	Weight	Std. Mean Difference IV, Random, 95% CI
Jancovic 1996	-2.75	4.47	13	-3.18	4.02	11	49.4%	0.10 [-0.71, 0.90]
Jog 2020	-1.92	1.61	19	-0.5	1.73	11	50.6%	-0.84 [-1.61, -0.06]
**Total (95% CI)**			**32**			**22**	**100.0%**	**-0.38 [-1.29, 0.54]**

Heterogeneity: Tau² = 0.27; Chi² = 2.68, df = 1 (P = 0.10); I² = 63%
Test for overall effect: Z = 0.80 (P = 0.42)

Figure S2. Effect of Botulinum toxin on tremor in patients with essential tremor anatomical-guidance.

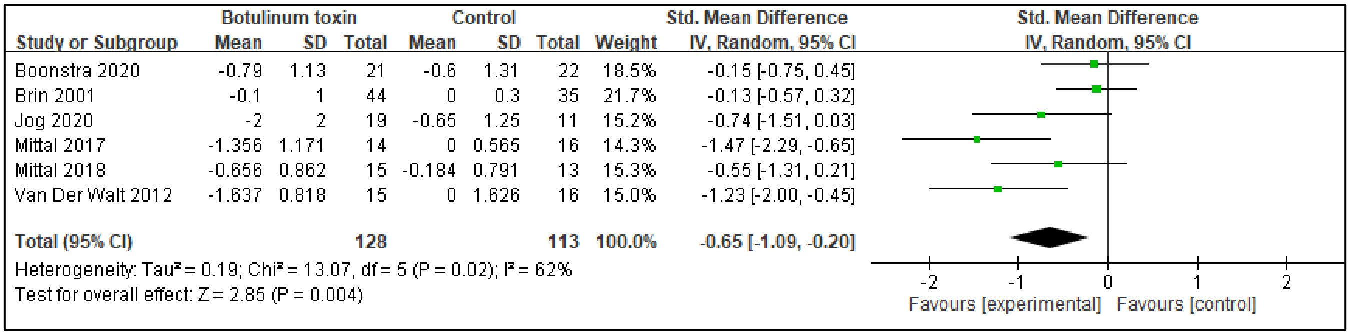

Study or Subgroup	Botulinum toxin Mean	SD	Total	Control Mean	SD	Total	Weight	Std. Mean Difference IV, Random, 95% CI
Boonstra 2020	-0.79	1.13	21	-0.6	1.31	22	18.5%	-0.15 [-0.75, 0.45]
Brin 2001	-0.1	1	44	0	0.3	35	21.7%	-0.13 [-0.57, 0.32]
Jog 2020	-2	2	19	-0.65	1.25	11	15.2%	-0.74 [-1.51, 0.03]
Mittal 2017	-1.356	1.171	14	0	0.565	16	14.3%	-1.47 [-2.29, -0.65]
Mittal 2018	-0.656	0.862	15	-0.184	0.791	13	15.3%	-0.55 [-1.31, 0.21]
Van Der Walt 2012	-1.637	0.818	15	0	1.626	16	15.0%	-1.23 [-2.00, -0.45]
**Total (95% CI)**			**128**			**113**	**100.0%**	**-0.65 [-1.09, -0.20]**

Heterogeneity: Tau² = 0.19; Chi² = 13.07, df = 5 (P = 0.02); I² = 62%
Test for overall effect: Z = 2.85 (P = 0.004)

Figure S3. Effect of Botulinum toxin on hands tremor between 7–12 weeks.
